# Optical Genome Mapping Reveals Complex and Cryptic Rearrangement Involving *PML*::*RARA* Fusion in Acute Promyelocytic Leukemia

**DOI:** 10.3390/genes15111402

**Published:** 2024-10-30

**Authors:** Melanie Klausner, Victoria Stinnett, Jen Ghabrial, Laura Morsberger, Natalie DeMetrick, Patty Long, Jing Zhu, Kirstin Smith, Trisha James, Emily Adams, Ying S. Zou

**Affiliations:** Department of Pathology, Johns Hopkins University School of Medicine, Baltimore, MD 21287, USA; mhardy22@jhmi.edu (M.K.); vlloyd3@jhmi.edu (V.S.); jghabri1@jh.edu (J.G.); lmorsber@jhmi.edu (L.M.); nescola1@jhu.edu (N.D.); plong@jhmi.edu (P.L.); jzhu23@jh.edu (J.Z.); ksmith83@jhmi.edu (K.S.); tjames17@jhmi.edu (T.J.); eadams32@jhmi.edu (E.A.)

**Keywords:** OGM optical genome mapping, APL acute promyelocytic leukemia, *PML*, *RARA*, cryptic, insertion, FISH, karyotype

## Abstract

**Background/objectives:** Acute promyelocytic leukemia (APL) is an aggressive subtype of acute myeloid leukemia (AML), characterized by the hallmark translocation t(15;17) resulting in a *PML*::*RARA* fusion. Once diagnosed, APL is now considered to be one of the most treatable forms of AML. However, without early detection and treatment, the disease is associated with rapid deterioration and lethal side effects. **Methods:** We describe a case of diagnostic APL presenting with a normal karyotype, normal *RARA* break-apart FISH, and unclear, atypical *PML/RARA* FISH findings. We used optical genome mapping (OGM) to characterize this atypical *PML/RARA* fusion. **Results:** OGM allowed for detection of a *PML*::*RARA* fusion resulting from a cryptic and complex insertion of *PML::RARA* into *RARA* on 17q21.2 whereby a segment of 15q24.1 was inserted into the 17q21.2. The recipient breakpoint of the insertion was at intron 2 of the *RARA* gene and the donor breakpoint of the insertion was at exon 5/intron 6 of the *PML* gene. **Conclusions:** This is the first report of an insertional *PML*::*RARA* fusion into the *RARA* gene on 17q detected by OGM. OGM has demonstrated its utility in a clinical cytogenetics environment, allowing for clearer characterization and diagnosis of various neoplasms.

## 1. Introduction

Acute promyelocytic leukemia (APL) is a subtype of acute myelocytic leukemia (AML), also known as AML-M3, accounting for approximately 10% to 15% of newly diagnosed AML cases per year [1,2]. APL is characterized by a block in the differentiation of leukemic cells, halting at the promyelocyte stage, and is associated with a signature chromosomal abnormality t(15;17)—a translocation between the long arm of chromosomes 15 and 17 resulting in the fusion of the *PML* (promyelocytic leukemia) and *RARA* (retinoic acid receptor α) genes. In addition to blocking differentiation, expression of the resulting *PML*::*RARA* fusion protein prevents apoptosis, allowing for neoplastic proliferation [2].

Prior to the introduction of all-trans retinoic acid (ATRA) and arsenic trioxide (ATO), APL was one of the fatal forms of AML, often the result of intracranial or pulmonary hemorrhage resulting from APL-induced bleeding disorders [3]. However, for patients who survive induction, up to 90% will see long-term remission with the current standard of care [3]. Because the rapid initiation of these treatments often dictates the disease course for APL patients, early detection is paramount for overall survival. 

Optical genome mapping (OGM) is an emerging technology that has proved useful in clinical cytogenetics setting. The current standard of care in cancer cytogenetics includes karyotype metaphase analysis, FISH (fluorescent *in situ* hybridization), and chromosomal microarray (CMA). Karyotype allows for a whole-genome, single-cell description of the neoplasm, albeit at a relatively low resolution, typically less than 15 Mbp in an oncology setting. FISH provides rapid results for targeted analysis but can perform poorly for identifying or describing atypical abnormalities due to the highly specific curation of the FISH probes. CMA allows for the detection of much smaller genome-wide abnormalities that may be below the resolution of other techniques, but cannot identify balanced rearrangements, such as translocations and inversions. OGM allows for a whole-genome characterization at high resolution, sensitivity, and specificity, allowing for broader analysis within a single assay. 

Herein, we describe a case of APL that presented with a normal karyotype, normal *RARA* break-apart FISH, and atypical *PML*/*RARA* FISH findings. OGM revealed a cryptic *PML*::*RARA* fusion resulting from a complex insertion of *PML* into *RARA*, supporting the clinical utility of OGM as a cytogenetic technology. 

## 2. Materials and Methods

### 2.1. Standard of Care Cytogenetic Testing

Conventional G-banded chromosome studies were performed using standard techniques. A minimum of 20 metaphase cells were analyzed from a bone marrow specimen collected in sodium heparin tubes. Karyotyping was achieved through conventional G-banded chromosome analysis performed on unstimulated 24- and 48-h cultures. The abnormal karyotypes were described using the International System for Human Cytogenetic Nomenclature (2020) [4]. 

Fluorescence in situ Hybridization (FISH) was performed on interphase nuclei from a direct cultured using disease-specific panels of probes, according to the manufacturer’s protocol, as previously described [5,6]. The FISH panel included break-apart probes of *RARA* (15q) and *CBFB* (16q), dual color dual fusion *PML/RARA* probe set, dual color dual fusion *ETO/AML* probe set, and an AML/MDS FISH panel with probes 5p15.2 (D5S23, D5S721), 5q31 (*EGR1*), 7cen (D7Z1), 7q31 (D7S522), 8cen (D8Z2), 11q23 (*MLL*), 20q12 (D20S108), and 20q13.12 (D20S150) (Abbott Molecular, Inc., Des Plaines, IL, USA). A total of 200 nuclei per probe were visually evaluated with fluorescence microscopy by two technologists scoring separately using a Zeiss Axioscope system (Carl Zeiss Microscopy, LLC, Oberkochen, Germany), blinded from each other. The analysis was performed using CytoVision software, version 7.7. The specimen was considered abnormal if the results exceeded the laboratory-established cutoff for each probe set. Cutoff values were calculated during the clinical laboratory validation for each probe set, based on the probability of a false positive as calculated by the binomial distribution utilizing the β inverse function set to 95% confidence level. For *RARA* break apart FISH, the normal cutoff is 5% for 200 nuclei analyzed; for *PML*::*RARA* dual fusion FISH, the normal cutoff is 1.5% for 200 nuclei analyzed.

Whole-genome SNP microarray was performed with DNA extracted from bone marrow, as previously described [5,7]. DNA was isolated from bone marrow aspirate using the Qiagen QIAamp protocol according to the manufacturer’s recommendations (QIAGEN, Germantown, MD, USA). Chromosomal microarray was performed using the whole genome Human Infinium CytoSNP-850K v1.3 BeadChip kit and protocol (Illumina, San Diego, CA, USA). SNP analysis was performed using both the KaryoStudio (version 1.4.3.0) and GenomeStudio (version v2011.1) softwares to examine B-allele frequency and LogR signal intensities to identify potentially pathogenic regions with genomic imbalance (Illumina, San Diego, CA, USA). 

### 2.2. Optical Genome Mapping

Optical genome mapping was performed on the fresh bone marrow specimen within 24 h of collection, as described previously [8,9]. Ultra-high molecular weight genomic DNA, UHMW gDNA, was isolated utilizing a Bionano SP-G2 Bone Marrow Aspirate (BMA) DNA Isolation Kit (Bionano Genomics, San Diego, CA, USA). In brief, the bone marrow was filtered and red blood cells were lysed before white blood cells were counted using the HemoCue WBC System, allowing for approximately 1.5 million white blood cells to proceed to DNA isolation (HemoCue America, Brea, CA, USA). Thermolabile Proteinase K was utilized for cell lysis and digestion was halted via heat inactivation. gDNA was separated from the solution using a magnetic nanodisk, washed, and subsequently unbound from the disk via an Elution Buffer, mixing the sample throughout the process with a HulaMixer Sample Mixer (Thermo Fisher Scientific, Waltham, MA, USA). Broad Range Qubit quantification was performed, after overnight homogenization of the DNA, allowing for approximately 750 ng of UHMW gDNA to proceed into labeling using a Bionano Direct Label and Stain-G2 (DLS-G2) Kit (Thermo Fisher Scientific, Waltham, MA, USA). The direct labeling enzyme, DLE-1, fluorescently labels the UHMW gDNA at specific motif sites, CTTAAG, without the introduction of nicks. After a dye clean-up step to remove excess labeling, the DNA was stained and quantified using High Sensitivity Qubit to ensure adequate DNA was present. The sample was loaded onto a single flow cell of a G3.3 Saphyr Chip and run on a Saphyr instrument, establishing the target DNA collection at 1500 Gbp. 

Saphyr run metrics were evaluated via a Bionano-generated Molecule Quality Report (MQR), shown in Table 1. The MQR metrics passed our laboratory-established QC, allowing for submission of the Rare Variant Analysis pipeline through Bionano Solve version 3.8.2 against reference hg38. Analysis was performed using Bionano Access version 1.8.2. Recommended confidence filter settings were applied for all SVs, CNV, and aneuploidy calls. 

### 2.3. Molecular Testing for PML::RARA Fusions

Real-time quantitative RT-PCR (qRT-PCR) was performed in a CLIA/CAP-certified molecular diagnostics lab. RNA was extracted from bone marrow using standard procedures (Qiagen, Germantown, MD, USA). RNA was reverse transcribed using High-Capacity cDNA Reverse Transcription reagents (ABI), and fusion gene mRNA was detected by real-time PCR using the Qiagen PML-RARA Fusion Quant kits on a TaqMan 7900 or QuantStudio 6 instrument. A control mRNA (ABL1) was amplified to monitor the quality of the sample. In acute promyelocytic leukemia (APL), the Qiagen Fusion Quant PML-RARa bcr 1 (L form), PML-RARa bcr2 (V form), and PML-RARa bcr3 (S form) assays detected and quantified a t(15;17) translocation. For a NanoString gene fusion panel, 300 ng of RNA from the bone marrow specimen was used for the NanoString gene fusion assay in the present study following the manufacturer’s protocol, as previously described [6].

### 2.4. Targeted Next-Generation Sequencing (NGS) Mutation Assay

NGS was performed in CLIA/CAP-certified molecular diagnostics labs on a bone marrow specimen, as previously described [5,7]. DNA concentration was assessed using the Qubit fluorometer (Thermo Fisher Scientific, Waltham, MA, USA). Library preparation was performed using Kapa Roche (Wilmington, MA, USA) reagents, hybrid capture was performed using Integrated DNA Technologies probes (Coralville, IA, USA), and products were sequenced using NovaSeq (paired-end technology; Illumina, San Diego, CA, Coralville, IA, USA). The targeted NGS assay used 40,670 Integrated DNA Technologies probes; for a list of covered cancer genes in the targeted NGS assay, see Supplementary Table S1. Analysis was performed using human reference sequence genome assembly hg19 (NCBI build GRCh37/hg19). An in-house variant caller software, as described previously, was used to generate gene variants/mutations from the targeted NGS data [5,6,7,10]. NGS had coverage (>250×) and mutant allele frequency (>5%).

## 3. Results

### 3.1. Clinical History

**Figure 1:**
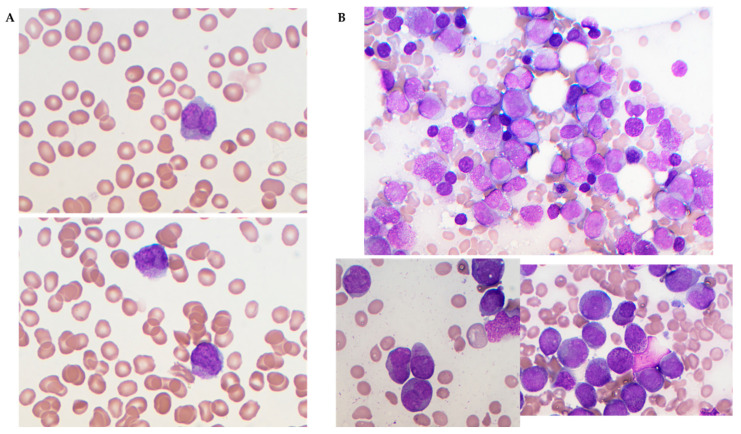
Peripheral blood and bone marrow smears. (**A**) Peripheral blood smears were pancytopenic with circulating blasts, large promyelocytes (120× objective). (**B**) Bone marrow smears showed hypercellularity with abnormal large promyelocytes with a prominent nucleus and heavily granulated cytoplasm (Auer rods were occasionally observed). The top image was bone marrow aspirate at 60× (objective) and the below images were bone marrow aspirate at 120× (objective).

This case study follows a 71-year-old female, with prior cervical cancer resulting in partial hysterectomy, who presented with spontaneous bruising and was found to be pancytopenic with circulating blasts (Figure 1). Peripheral blood coagulation studies indicated high D-Dimer (11.01 mg/L FEU) and low fibrinogen (101 mg/dL), and flow cytometry displayed high side scatter and autofluorescence with bright CD33 and negative HLA-DR (see Supplementary Table S2 for full lab test results). Based on these initial findings, treatment for acute promyelocytic leukemia was rapidly initiated with ATRA/ATO. Bone marrow biopsy was performed and sent for karyotype, FISH, and NGS testing.

### 3.2. Standard of Care Cytogenetic Findings

**Figure 2:**
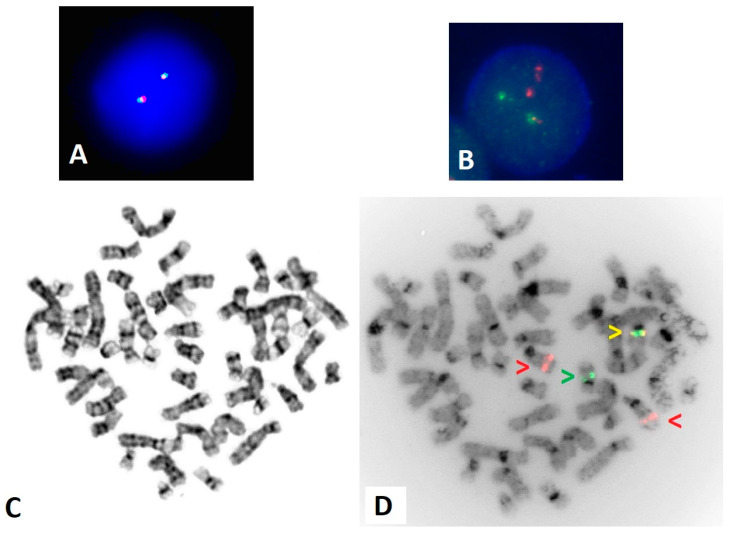
FISH using the *RARA* break apart and *PML*/*RARA* dual fusion probes. (**A**) Interphase FISH for *RARA* break apart displaying a normal 2F pattern. (**B**) Interphase for *PML*/*RARA* dual fusion displaying an atypical abnormal pattern of 2R 1G 1F. (**C**) G-banded metaphase displaying a normal female karyotype. (**D**) Inverted DAPI image of metaphase *PML*/*RARA* FISH, with red arrows indicating *PML* on 15q24, a green arrow indicating *RARA* on 17q21, and a yellow arrow indicating a fusion. The *PML*::*RARA* gene fusion is present on a normal-appearing 17q.

Conventional chromosome analysis displayed a normal female karyotype in all twenty metaphase cells analyzed. Interphase FISH for *PML*/*RARA* dual fusion and *RARA* break apart were expedited based on the clinical indication. FISH for *RARA* break apart was found to be within normal limits with a pattern of 2F, representing two fusion or two seemingly intact copies of *RARA* (Figure 2A). However, the *PML*/*RARA* dual fusion FISH displayed a high percentage of an atypical fusion pattern, 2R 1G 1F, where red (R) hybridizes to *PML* on 15q24 and green (G) hybridizes to *RARA* on 17q21, while a fusion (F) indicates overlap of these FISH gene regions, the result of a gene fusion event (Figure 2B).

Follow-up metaphase FISH was utilized to confirm and clarify the atypical interphase FISH findings. Metaphase FISH for *RARA* break apart probes confirmed the two fusions were present on the 17q regions (Supplementary Figure S1). Metaphase FISH for *PML*/*RARA* dual fusion probes confirmed a fusion signal on a normal 17q according to G-banded chromosome analysis (Figure 2C,D). 

### 3.3. Optical Genome Mapping for Complex Rearrangements of PML and RARA Genes

**Figure 3:**
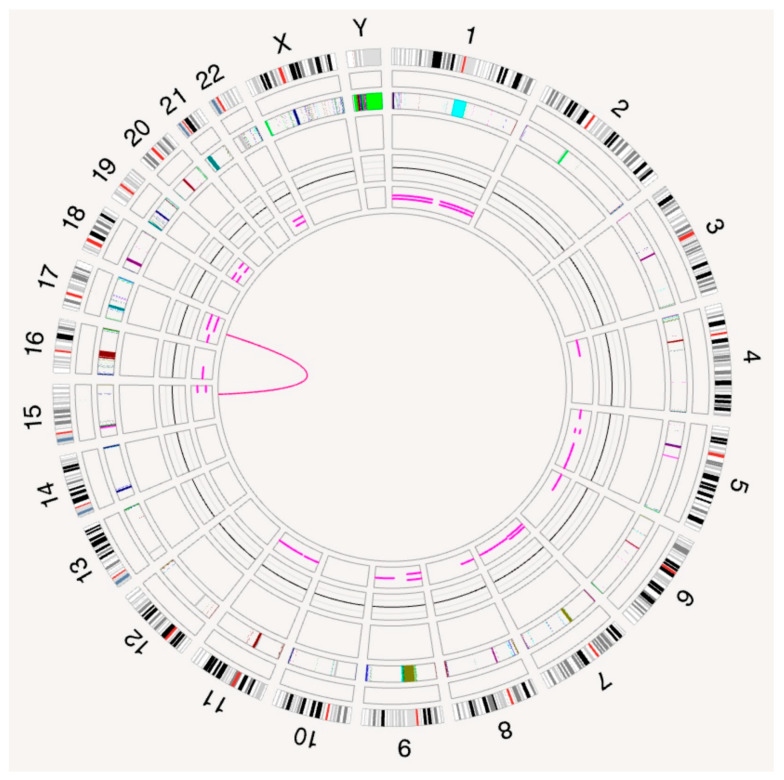
The OGM circos plot, displaying a rearrangement between chromosomes 15 and 17 (pink line in the center). Circos tracks starting from outer rings show a cytoband track, targeted gene track, masked region track, structural variant (SV) track, copy number variant (CNV) track, variant allele frequency (VAF) segment track, and the central translocation track.

The OGM circos plot displayed a translocation between the long (q) arms of chromosomes 15 and 17 (Figure 3). 

**Figure 4:**
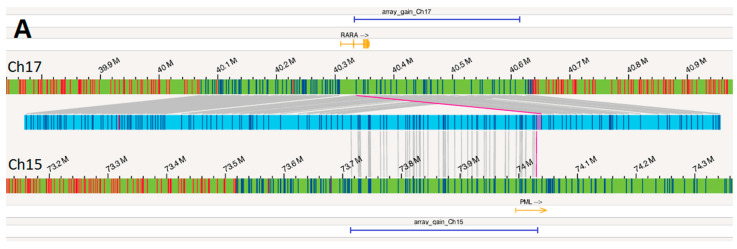

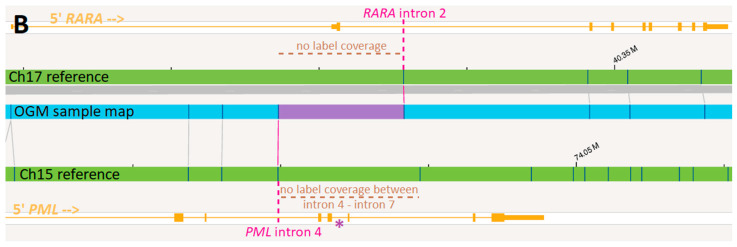
(**A**) The OGM map (light blue horizontal bar) between the reference for chromosome 17 (green bar above the OGM map) and reference for chromosome 15 (green bar below the OGM map) where the dark blue vertical lines on the OGM map indicate OGM labels mapping to the two references as indicated by the gray lines. The *RARA* gene is shown in orange above the chromosome 17 reference and the *PML* gene in orange is shown below the chromosome 15 reference. Regions of gain identified by CMA are shown by horizontal dark blue bars. (**B**) Zooming into the rearrangement occurring in *PML* and *RARA* (solid pink line in Figure 4A), OGM provides breakpoints within intron 2 of *RARA* and intron 4 of *PML* (pink dotted lines). Due to lack of label coverage (brown dotted lines), the actual breakpoint within *PML* could have potentially occurred anywhere between intron 4 and intron 7. *PML* bcr1 at intron 6 (purple asterisk) was described as the fusion site by RT-PCR.

Examination of this OGM call revealed a complex rearrangement event resulting from the insertion of 15q material into 17q (Figure 4A). Approximately 320 kbp of 15q material, including a majority of the *PML* gene, was inserted into 17q, within the *RARA* gene. One of the two breakpoints bordering the insertion resulted in the *PML*::*RARA* fusion (Figure 4B). 

**Figure 5:**
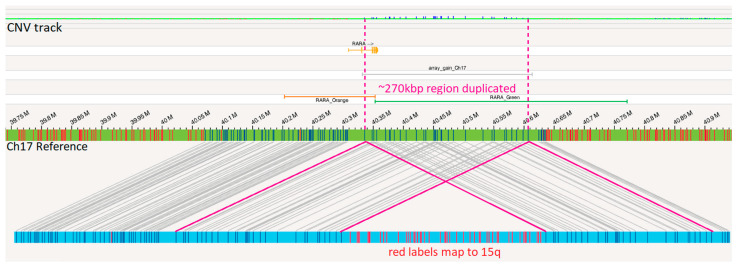
Duplication of 17q: the OGM map (light blue horizontal bar) below the reference for chromosome 17 (green bar) where the dark blue vertical lines on the OGM map indicate OGM labels mapping to the chromosome 17 reference and the red vertical lines on the OGM map indicate OGM labels mapping to the chromosome 15 reference. The *RARA* gene is shown in orange above the chromosome 17 reference. Region of gain detected by microarray is shown with silver bar. Pink lines show how two segments of the OGM map cover the same region of 17q, resulting in copy number gain. The OGM CNV track at the top displays CN gain (small vertical blue bars) in this region of duplication. “RARA_Orange” and “RARA_Green” indicate the regions covered by the orange/red and green portions of the *RARA* break apart FISH probe.

Additionally, there is a duplication of approximately 270 kbp of 17q material, including the 3′ end of the *RARA* gene (covering exons 3–9), supported by corresponding copy number (CN) gain over these labels by OGM (Figure 5). OGM labels spanning the region of 15q inserted also had CN gain (covering at least exons 1–4 of the *PML* gene). 

### 3.4. Molecular and Chromosomal Microarray Findings

The gene fusion assay and the qRT-PCR assay revealed the presence of a *PML*::*RARA* long fusion transcript. NGS detected somatic mutations in *WT1* (*c.1113+1G>C*) and *FLT3* (*p.D839A*). 

CMA revealed a 319 kilobase (kb) gain on 15q24.1 involving exons 1 to 5 of the *PML* gene (ending within exon 5; NM_033238), arr[GRCh38] 15q24.1(73,713,259–74,032,683) × 3, and a 282 kb gain on 17q21.2 including intron 2 to exon 9 of the *RARA* gene (starting within intron 2; NM_000964), arr[GRCh38] 17q21.2(40,332,287–40,614,034) × 3. These gains were shown with dark blue horizontal bars at the top and bottom of Figure 4 and in Supplementary Figure S2.

**Figure 6:**
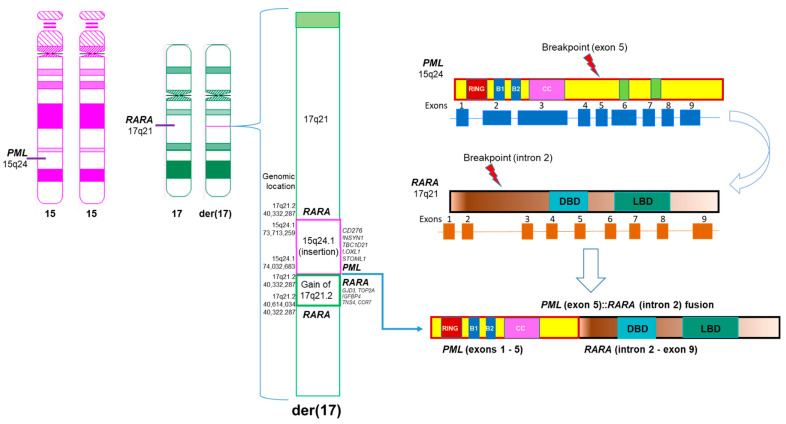
Diagram of the complex insertional *PML*::*RARA* fusion based on OGM and other genetic data in this study. Left: Ideograms of 2 normal chromosomes 15, a normal chromosome 17, and a derivative chromosome 17 with a complex, submicroscopic, and insertional *PML*::*RARA* fusion. Middle: The derivative chromosome 17 had a *PML*::*RARA* fusion due to an insertion of 15q24.1 including *PML* and other genes and a tandem duplication of 17q21.2 including *RARA* gene and other genes. Right: Breakpoints involved in the insertional *PML*::*RARA* fusion.

Combination of OGM, molecular, and CMA findings provided comprehensive characterization of this complex insertional *PML*::*RARA* fusion in this case (Figure 6).

## 4. Discussion

The diagnosis of APL relies on the identification of *PML*::*RARA* fusion. A definitive and timely genetic diagnosis of *PML*::*RARA* fusion is critical for ATRA treatment and systemic therapy regimen decisions with inclusion of arsenic trioxide and/or chemotherapy in APL patients. While classic/typical balanced t(15;17) rearrangements are found in the majority (approximately 87% to 92%) of APL cases, up to 13% of patients may have atypical and cryptic results by standard-of-care cytogenetic tests [11,12]. In this study, we rapidly characterized a complex and submicroscopic insertional *PML::RARA* fusion by OGM. This is the first report of an insertional *PML*::*RARA* fusion along with a small tandem duplication of 17q21.2 detected by OGM. OGM has demonstrated its diagnostic utility in a clinical cytogenetics environment, allowing for clearer characterization and diagnosis of complex rearrangements.

### 4.1. Interpretation of Standard of Care and OGM Results

Presenting with a normal karyotype, normal *RARA* break apart FISH findings and an atypical single fusion FISH pattern with the *PML*/*RARA* dual fusion probe set. An OGM analysis was performed to achieve a clear characterization of a complex rearrangement leading to an insertional *PML*/*RARA* fusion, which was cryptic by standard-of-care karyotyping analysis and *RARA* break apart FISH. Furthermore, OGM provided a fast turn-around time of approximately 4 days and with an easy workflow, all of which are very useful and important for routine clinical practices.

OGM provided precise characterization of this complex rearrangement. OGM revealed two CN variants involving this rearrangement. OGM displays a region of 17q21.2 that maps to both ends of the insertion event, resulting in a split duplication of approximately 270 kbp of 17q21.2 material, beginning at the translocation site in *RARA* intron 2 and extending to beyond the end of *RARA* until the *CCR7* gene (Figure 6). Although both OGM and CMA revealed this CN gain of 17q21.2, only OGM revealed the order of this gain, which is a tandem duplication event. Note that the slight size discrepancy for the region of 17q21.2 gain as called by OGM and CMA could be due to probe design as well as intrinsic differences between assays. While both OGM and CMA also called the region of 15q24.1 that was inserted into 17q21.2 as a region of CN gain, only OGM revealed an insertional segment of 15q24.1 into 17q21.2 with more fundamental significant genomic information. Given the smaller size of these two CN gains and the coverage of FISH probes according to probe design, FISH may not reveal these gains. The normal *RARA* break apart FISH findings could be explained by the highly specific, low-resolution nature of the break apart FISH probes. With a break apart FISH probe size of nearly 600 kbp, the 320 kbp insertion and 270 kbp duplication went undetected (Figure 5, Supplementary Figure S1). 

A balanced translocation t(15;17) would result in the 5′ *PML*–3′ *RARA* transcript as well as the reciprocal 5′ *RARA*–3′ *PML* transcript. In APL, the 5′ *PML*–3′ *RARA* transcript is present in all cases, while the 5′ *RARA*–3′ *PML* transcript is present in approximately 75% of cases [13]. In nearly all *PML*::*RARA* fusions, the *RARA* breakpoint is within intron 2, while the *PML* breakpoint is within one of three known regions: bcr1 (intron 6), bcr2 (exon 6), or bcr3 (intron 3). These three variations result in the long variant, and short form of the fusion transcript, respectively. While OGM indicates a breakpoint within intron 4 of *PML*, it must be noted that OGM breakpoints rely on the presence of OGM labels, which are the six-base pair motif CTTAAG. Because the next OGM label does not occur until intron 7 of *PML*, it is reasonable to assume the *PML* breakpoint is somewhere between intron 4 and intron 7. In this case, RT-PCR was able to detect presence of the long form of the fusion transcript, corresponding to bcr1 in intron 6 on *PML*. However, due to the extremely specific nature of PCR primer designs, there have been reports of atypical *PML*::*RARA* fusions that went undetected by the conventional PCR methods [14,15]. Thus, although PCR results can be achieved faster than OGM results, the added benefit of OGM data and its potential abilities to unambiguously reveal atypical *PML*::*RARA* fusions, still achieved at a relatively low turn-around-time, cannot be ignored.

### 4.2. Clinical Implications

Only approximately 5% of APL cases are in patients older than 70 years of age [16]. Older adults with ATRA-treated APL have less favorable prognoses as compared to younger adults; it is unclear if this simply is the result of various aspects of advanced age, including increased incidence of comorbidities, or the result of an undefined biological characteristic of that cohort [17]. 

Presenting with pancytopenia, a low white blood cell count precludes categorization of this patient as having high-risk APL. Following the standard of care for non-high-risk APL–ATRA/ATO without additional cytotoxic chemotherapy—the thirty-day treatment course was complicated by differentiation syndrome, a common side-effect of ATRA/ATO therapy. Post-treatment bone marrow displayed no residual leukemia by flow cytometry; however, FISH for *PML*/*RARA* still displayed a high percentage of the same atypical signal pattern. Although APL is a well characterized subtype of AML, the implications of these cytogenetic findings are unclear. Further research is warranted to determine the diagnostic and prognostic significance of similar atypical abnormalities and the associated phenotype and response to treatment. 

## 5. Conclusions

This is the first report of an insertional *PML::RARA* fusion along with a small tandem duplication of 17q21.2 detected by OGM. Optical genome mapping has proven to be a useful tool to aid in the diagnostic cytogenetic interpretation for this case of APL. In conjunction with the standard of care methods, OGM will allow for a more detailed description of various neoplasms with the goal of further improving the personalized treatments available for these diseases. 

## Figures and Tables

**Table 1:** Molecule Quality Report (MQR).

Metric	Ideal/Target Value	Actual Value
Total DNA (≥150 kbp)	1500 Gbp	1550.38 Gbp
N50 (≥150 kbp)	>230 kbp	376.5 kbp
Average Label Density (≥150 kbp)	14–17 labels/100 kbp	15.81 labels/100 kbp
Effective Coverage	>350	450.96
Map Rate	>70%	93.1%

## Data Availability

The dataset for the current study are available from the corresponding author upon reasonable request.

## Supplementary Materials

The following supporting information can be downloaded at: https://www.mdpi.com/article/10.3390/genes15111402/s1, Supplementary Table S1: Genes covered in NGS panel. Table S2: Lab test results of CBC and bone marrow. Supplementary Figure S1: Metaphase FISH for *RARA* break apart; Figure S2: Chromosomal microarray assay (CMA) results.
