# Supplementary material for: Optical Genome Mapping Reveals Complex and Cryptic Rearrangement Involving PML::RARA Fusion in Acute Promyelocytic Leukemia
Source: Genes (Basel). 2024 Oct 30;15(11):1402. doi: 10.3390/genes15111402 (PMC11594156; doi:10.3390/genes15111402)
Supplement: Supplementary file 1 [file genes-15-01402-s001.zip › genes-3262842-supplementary.pdf]

Supplemental materials include Supplemental Figure S1-S2 and Supplemental Table S1-S2

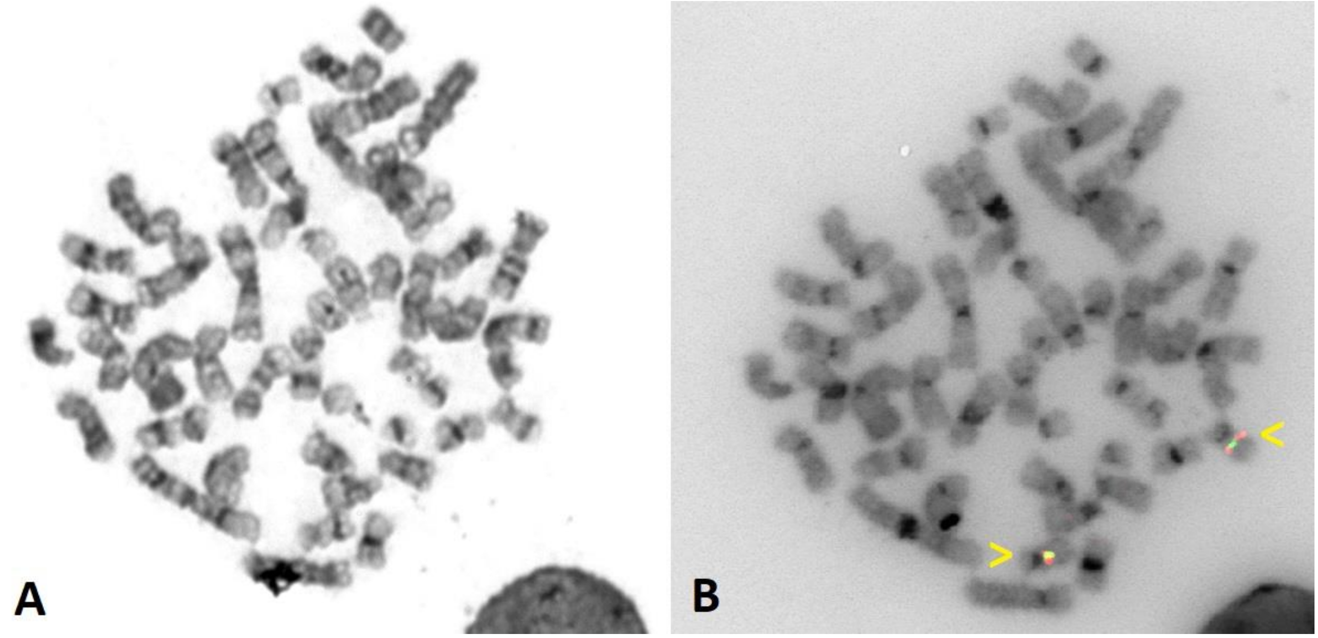

Supplementary Figure S1. Metaphase FISH for RARA break apart, demonstrating the two in-tact fusion signals are present on the two copies of chromosome 17. **A:** G-banded metaphase displaying a normal female karyotype. **B:** Inverted DAPI image of metaphase RARA FISH, with yellow arrows indicating fusions.

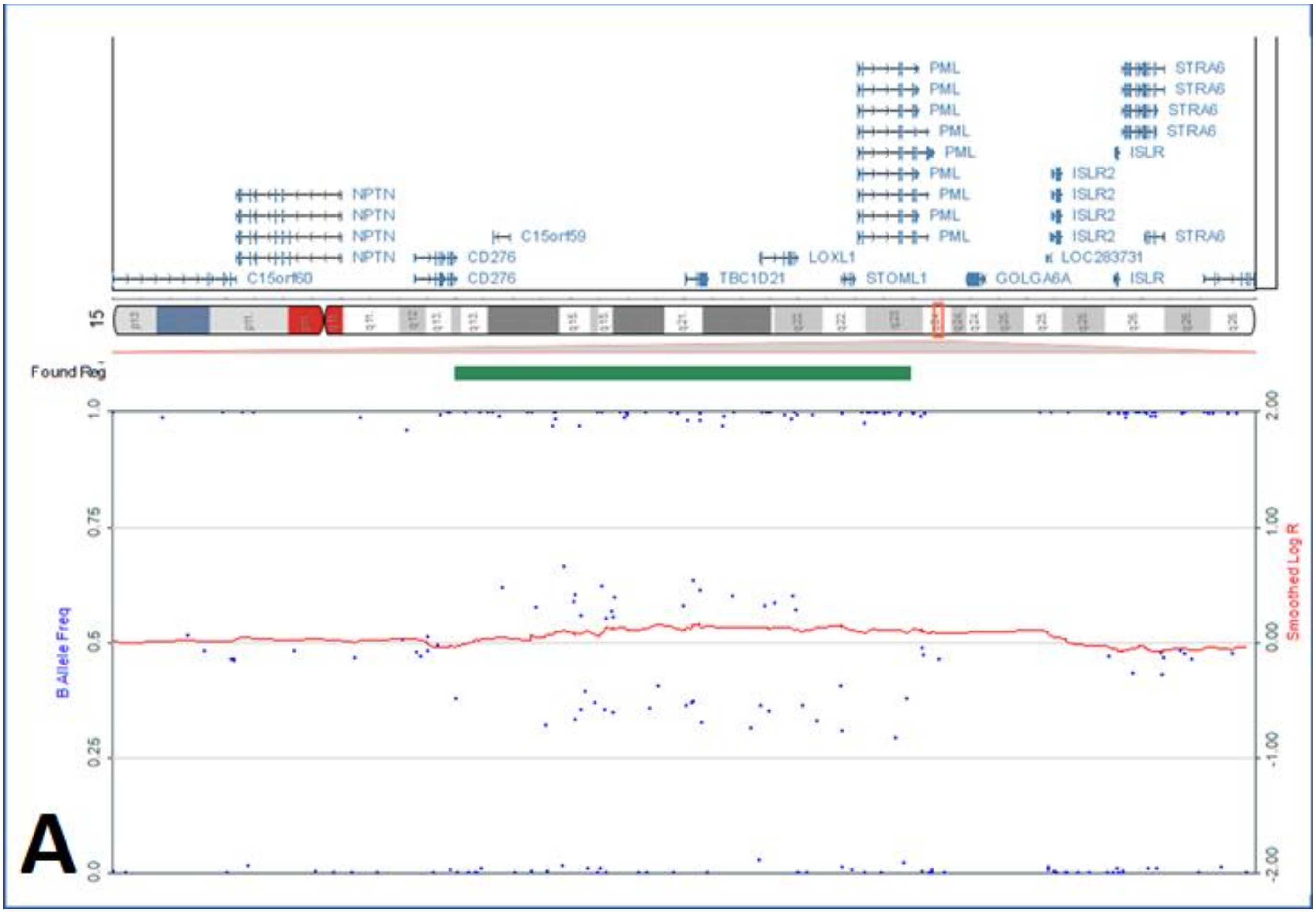
PML
STRA6
ISLR
ISLR2
NPTN
C15orf59
CD276
LOXL1
LOC283731
C15orf60
TBC1D21
STOMIL1
GOLGA6A
15
Found Reg
B Allele Freq
Smoothed Log R
1.0
0.75
0.5
0.25
0.0
2.00
1.00
0.00
-1.00
-2.00
A

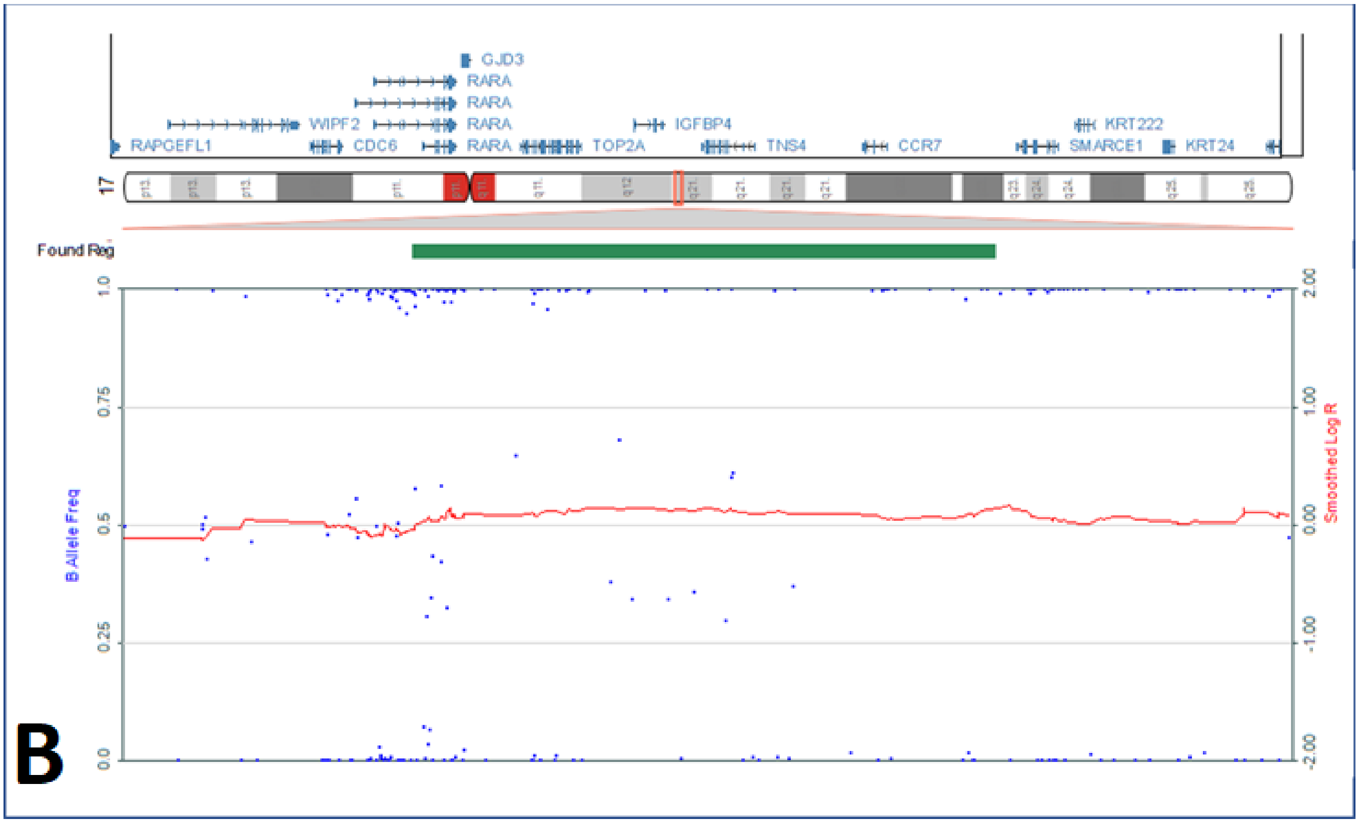

Supplementary Figure S2: SNP microarray plots displaying genotype information using the B-allele frequency data (blue dots) and the intensity data for the average LogR value (red line). The horizontal green bar indicates the regions of gain identified by microarray which are demonstrated by an increase in intensity data as well as the absence of heterozygous genotypes noted by the B-allele frequency data. Specifically, microarray showed **A:** 319 kilobase gain on 15q24.1 within the *PML* gene (ending within exon 5, NM_033238, arr[GRCh38] 15q24.1(73,713,259-74,032,683)x3) and **B:** a 282 kilobase gain on 17q21.2 within the *RARA* gene (starting within intron 2; NM_000964, arr[GRCh38] 17q21.2(40,332,287-40,614,034)x3).

Supplemental Table S1: list of covered cancer genes in the targeted NGS assay

**ABL1**	NM_005157	**NF1**	NM_000267
**ASXL1**	NM_015338	**NFE2**	NM_006163
**ATM**	NM_000051	**NLRP1**	NM_033004
**ATRX**	NM_000489	**NOTCH1**	NM_017617
**BCL2**	NM_000633	**NOTCH2**	NM_024408
**BCL6**	NM_001706	**NPM1**	NM_002520
**BCOR**	NM_017745	**NRAS**	NM_002524
**BCORL1**	NM_021946	**NSD1**	NM_022455
**BRAF**	NM_004333	**NUP98**	NM_016320
**BTK**	NM_000061	**PAX5**	NM_016734

**CALR**	NM_004343	**PDGFRA**	NM_006206
**CARD11**	NM_032415	**PHF6**	NM_032458
**CBL**	NM_005188	**PIGA**	NM_002641
**CBLB**	NM_170662	**PIM1**	NM_001243186, NM_002648
**CD79A**	NM_001783	**PLCG2**	NM_002661
**CD79B**	NM_000626	**POT1**	NM_015450
**CDKN2A**	NM_000077	**PPM1D**	NM_003620
**CEBPA**	NM_004364	**PRDM1**	NM_001198
**CHEK2**	NM_007194	**PTEN**	NM_000314
**CREBBP**	NM_004380	**PTPN11**	NM_002834
**CSF3R**	NM_156039	**RAD50**	NM_005732
**CXCR4**	NM_001008540	**RECQL4**	NM_004260
**DDX41**	NM_016222	**RHOA**	NM_001664
**DNMT3A**	NM_022552	**RHOH**	NM_004310
**EP300**	NM_001429	**RUNX1**	NM_001754,
**ERBB2**	NM_004448	**SAMD9**	NM_017654
**ETV6**	NM_001987	**SAMD9L**	NM_152703
**EZH2**	NM_004456	**SETBP1**	NM_015559
**FAS**	NM_000043	**SF3B1**	NM_012433
**FBXW7**	NM_033632	**SGK1**	NM_005627
**FLT3**	NM_004119	**SRSF2**	NM_003016
**FOXO1**	NM_002015	**STAG2**	NM_006603
**GATA1**	NM_002049	**STAT3**	NM_139276
**GATA2**	NM_032638	**STAT5B**	NM_012448
**GNA13**	NM_006572	**STAT6**	NM_003153
**GNAS**	NM_000516	**TERT promoter**	NM_198253
**IDH1**	NM_005896	**TET2**	NM_001127208
**IDH2**	NM_002168	**TNFAIP3**	NM_001270507
**IKZF1**	NM_006060	**TNFRSF14**	NM_003820
**JAK2**	NM_004972	**TP53**	NM_000546
**KDM2B**	NM_032590	**U2AF1**	NM_006758
**KDM6A**	NM_021140	**UBA1**	NM_003334
**KIT**	NM_000222	**WT1**	NM_024426
**KMT2A**	NM_001197104	**ZRSR2**	NM_005089
**KMT2D/MLL2**	NM_003482		
**KRAS**	NM_004985		
**MEF2B**	NM_001145785		
**MPL**	NM_005373		
**MYC**	NM_002467		
**MYD88**	NM_002468		

Supplemental Table S2: key lab test results of peripheral blood and bone marrow aspirate

**Blood Results**		
D-Dimer (mg/L FEU)	11.01	H
Fibrinogen, Quant., Superstat (mg/dL)	101	L

INR, Protime	1.5	H
Prothrombin Time (sec)	15.3	H
APTT (sec)	25.2	
White Blood Cell Count (K/cu mm)	4.02	L
Red Blood Cell Count (M/cu mm)	2.21	L
Hemoglobin (g/dL)	7.2	L
Hematocrit %	21.1	L
Mean Corpuscular Volume (fL)	95.5	
Mean Corpus Hgb (pg)	32.6	
Mean Corpus Hgb Conc (g/dL)	34.1	
Mean Platelet Volume (fL)	12.1	
RBC Distribution Width (%)	15.8	H
Platelet Count (K/cu mm)	34	L
Nucleated RBC Number (K/cu mm)	0	
Neutrophils %	16	L
Lymphocyte %	11	L
Monocyte %	3	
Eosinophil %	1	
Basophil %	0	
Myelocyte %	1	H
Blasts %	46	HH
Promyelocyte %	20	H
ANC-Absolute Neut Count (K/cu mm)	0.64	L
Lymphocytes Absolute,Manual (K/cu mm)	0.44	L

L = Data is abnormally low

H = Data is abnormally high

HH = Data is critically high

**Bone Marrow Results**		
Plasma Cells %	0	
Blast %	0.5	
Promyelocytes %	84.5	H
Myelocytes %	1.5	L
Metamyelocytes %	0	L
Bands %	0	L
Neutrophils %	1	L
Lymphocytes %	4	L
Monocytes %	0	
Eosinophils %	0.5	L
Basophils %	2	H
Early Erythroid Precursors %	0	

Late Erythroid Precursors %	6	
Histiocytes %	0	L
Others %	0	
Megakaryocytes	Absent	
Cellularity	Normocellular	
Stromal Elements	Absent	
Myeloid Erythroid Ratio	15	
Comments, Bone Marrow: 200 nucleated cells identified on 2 coverslips scanned.		

L = Data is abnormally low

H = Data is abnormally high
